# Raman Spectroscopy: A Potential Diagnostic Tool for Oral Diseases

**DOI:** 10.3389/fcimb.2022.775236

**Published:** 2022-02-04

**Authors:** Yuwei Zhang, Liang Ren, Qi Wang, Zhining Wen, Chengcheng Liu, Yi Ding

**Affiliations:** ^1^ State Key Laboratory of Oral Diseases, National Clinical Research Center for Oral Diseases, Department of Periodontics, West China Hospital of Stomatology, Sichuan University, Chengdu, China; ^2^ State Key Laboratory of Oral Diseases, National Clinical Research Center for Oral Diseases, Department of Prosthodontics, West China Hospital of Stomatology, Sichuan University, Chengdu, China; ^3^ College of Chemistry, Sichuan University, Chengdu, China

**Keywords:** Raman spectroscopy, oral microbiota, dental caries, periodontitis, oral cancer

## Abstract

Oral diseases impose a major health burden worldwide and have a profound effect on general health. Dental caries, periodontal diseases, and oral cancers are the most common oral health conditions. Their occurrence and development are related to oral microbes, and effective measures for their prevention and the promotion of oral health are urgently needed. Raman spectroscopy detects molecular vibration information by collecting inelastic scattering light, allowing a “fingerprint” of a sample to be acquired. It provides the advantages of rapid, sensitive, accurate, and minimally invasive detection as well as minimal interference from water in the “fingerprint region.” Owing to these characteristics, Raman spectroscopy has been used in medical detection in various fields to assist diagnosis and evaluate prognosis, such as detecting and differentiating between bacteria or between neoplastic and normal brain tissues. Many oral diseases are related to oral microbial dysbiosis, and their lesions differ from normal tissues in essential components. The colonization of keystone pathogens, such as *Porphyromonas gingivalis*, resulting in microbial dysbiosis in subgingival plaque, is the main cause of periodontitis. Moreover, the components in gingival crevicular fluid, such as infiltrating inflammatory cells and tissue degradation products, are markedly different between individuals with and without periodontitis. Regarding dental caries, the compositions of decayed teeth are transformed, accompanied by an increase in acid-producing bacteria. In oral cancers, the compositions and structures of lesions and normal tissues are different. Thus, the changes in bacteria and the components of saliva and tissue can be used in examinations as special markers for these oral diseases, and Raman spectroscopy has been acknowledged as a promising measure for detecting these markers. This review summarizes and discusses key research and remaining problems in this area. Based on this, suggestions for further study are proposed.

## 1 Introduction

Oral disease is a major global public health issue, with more than 3.5 billion people suffering from chronic and progressive oral diseases worldwide. Dental caries, periodontal diseases, and oral cancers are the most prevalent and serious oral diseases. For patients, these cause a serious burden both health-wise and financially, significantly degrading the quality of life (Peres et al., 2019). In 2015, the prevalence of untreated deciduous and permanent teeth was 7.8% and 34.1%, respectively (Kassebaum et al., 2017). Severe periodontitis has been considered the sixth most common infectious disease worldwide, with 10.8% of people, i.e., 743 million, being affected in 2010 (Kassebaum et al., 2014), which increased to 1.1 billion by 2019 (Chen et al., 2021). In 2018, 177,384 people died from lip and oral cancer (Bray et al., 2018). Oral diseases also impose a great economic burden. In 2015, the direct cost of oral diseases worldwide was 356.8 billion US dollars, and the indirect cost was 187.61 billion US dollars (Righolt et al., 2018). The oral diseases mentioned above are often not detected until tissue destruction has occurred. For caries and periodontitis, the destruction of enamel, dentin, and alveolar bone is irreversible, and delayed discovery and treatment can even lead to tooth loss, affecting pronunciation, masticatory function, and aesthetics. For oral cancer, the accuracy of early diagnosis and incision directly affects the recurrence and survival rates. The current clinical detection methods remain insufficient. In terms of caries, the identification of carious dentin and healthy dentin can reduce the loss of healthy tissue during lesions removal, reduce the damage and the possibility of tooth fracture. As for periodontitis, it is known that hyperglycemia, smoking, stress, etc. are risk factors for periodontitis. Patients with risk factors or previous used concept, aggressive periodontitis, are at higher risk of periodontitis, and destruction of periodontal tissues are faster. However, the effect of these factors in the progress of periodontitis in each individual cannot be found through the existing periodontal clinical examination. Local biomarkers like the expression of local subgingival flora virulence factors and the level of some inflammatory factors in gingival crevicular fluid can warn us the risk of disease progression, while the laboratory examination is time-consuming and requires more biological samples. Especially in large-scale epidemiological investigations, a new diagnostic tool can unify the detection standards and make the conclusions more scientific, therefore, it is more suitable for non-periodontal and non-endodontic specialist. For oral cancer, early malignant lesions are not easily diagnosed non-invasively and accurately. Delayed diagnosis makes the treatment more difficult and the prognosis is affected. Moreover, an intraoperative freezing section to determine whether the incision is clean requires a pathologist at a higher professional level. Therefore, methods for detecting lesions earlier and more sensitively could more easily avoid irreversible tissue destruction and improve the curative effect and prognosis, which are crucial to the prevention and treatment of oral diseases (Dillon et al., 2015). Raman spectra are inelastic scattering spectra based on the Raman effect, which are obtained using scattered light with a different frequency from the incident light (Raman CVK and S., 1928). Raman spectroscopy (RS) can provide fast, accurate, sensitive, and *in situ* detection analysis. It can sensitively and accurately reflect changes in material composition and structure. With the emergence of nanotechnology, advanced optical microscopes and miniaturized lasers have been developed, and problems such as weak signal, low signal-to-noise ratio, and strong autofluorescence background have also been mitigated to allow RS to be gradually applied in the biomedical field. RS has been used to detect bacteria and the compositions of cells, tissues, and biofluids, and in the past few decades it has received increasing interest for medical prognosis and diagnosis (Morris, 1999; Singh et al., 2016). It has emerged as a potential chairside microbiological diagnostic approach (Howell et al., 2011). There are obvious differences between composition and structure of dental caries and intact tooth tissues, and also between oral cancer lesions and healthy tissues. Therefore, the potential of RS in the diagnosis and prognosis of these two diseases is obvious. In the case of periodontitis, studies have shown that biomarkers in saliva can distinguish gingivitis from periodontitis (Gonchukov and Sukhinina, 2011; Hernández-Cedillo et al., 2019), and biomarkers in gingival crevicular fluid (GCF) including glycosaminoglycans and some inflammatory mediators, such as prostaglandin E_2_, can detect high-risk groups of periodontal disease (Curtis et al., 1989). The changes in the composition of subgingival flora and the expression of key pathogenic bacteria virulence factors indicate the advancement or relief of periodontitis. When the target substance is specific molecule, the key information required is concentrated to several chemical bonds of the target molecule, which can be more easily extracted from RS. It can be seen that RS has the potential to assist diagnosis and improve prognosis.

Recently, many studies have suggested that RS could assist in the diagnosis and prognostic evaluation of oral diseases, such as dental caries (Almahdy et al., 2012; Yang et al., 2014; Seredin et al., 2015; Toledano et al., 2015b; Rodrigues et al., 2017), periodontal diseases, and oral cancers (Krishna et al., 2004; Panikkanvalappil et al., 2013; Krishna et al., 2014; Sahu et al., 2015; Barroso et al., 2016; Malik et al., 2017; Mian et al., 2017; Barroso et al., 2018; Xue et al., 2018). Because minor changes in quantity can be detected by RS, it has been used in detecting early caries, finding the boundaries of dentin caries, and estimating the effects of drugs on the remineralization of decalcified tooth tissues (Carvalho et al., 2013; Milly et al., 2014; Yang et al., 2014; Adachi et al., 2015; Seredin et al., 2015; Toledano et al., 2015a; Toledano et al., 2015b; Kerr et al., 2016; Pezzotti et al., 2017; Rodrigues et al., 2017; Occhi-Alexandre et al., 2018). Interestingly, it has also been used to analyze multiple oral bacteria and differentiate saliva between patients with periodontal disease and healthy volunteers (HV) (Gonchukov et al., 2011; Kriem et al., 2020). Regarding oral cancers, many studies have focused on detecting and differentiating malignant, precancerous, benign, and normal tissues to assist diagnosis; finding an adequate surgical margin in an operation to improve the curative effect; and analyzing spectra to predict the recurrence risk (Krishna et al., 2004; Panikkanvalappil et al., 2013; Krishna et al., 2014; Sahu et al., 2015; Barroso et al., 2016; Malik et al., 2017; Mian et al., 2017; Barroso et al., 2018; Xue et al., 2018). In general, RS has the potential to assist in diagnosis and treatment as well as prognostic evaluation. The purpose of this review is to draw attention to the potential of RS in assisting clinical diagnosis and prognostic evaluation of caries, periodontitis, and oral cancer owing to its high potential for chairside detection. In this review, we focus on the application of RS to oral diseases and discuss problems that must be further explored.

## 2 Raman Spectroscopy

### 2.1 Principle of Raman Spectroscopy

C.V. Raman and his team discovered the Raman effect in 1928 (Raman CVK and S., 1928). They proposed that when ordinary light passes through a pure medium (water or gas), a small amount of scattered light, with frequency dissimilar to that of the incident beam, is produced. This phenomenon is called Raman scattering. This occurs because when a small number of photons collide with the chemical bonds in the sample, their energy is absorbed or lost, and the frequency of the scattered light changes accordingly. Although the frequency of Raman scattering light changes rather than being dependent on the incident light frequency, the shift is related to the molecular bonds. The bands in the Raman spectra are the specific manifestations of different molecular bonds in the sample, appearing as a unique spectral “fingerprint” of every substance (Krafft and Popp, 2015).

Although RS is widely applied in biomedical fields, it still has some shortcomings, including weak signal, low signal-to-noise ratio, and strong self-fluorescent background. With the advent of nanotechnology, advanced optical microscopes, fiber optics, and miniaturized lasers, these have been combined with RS to obtain stronger Raman signals. Surface-enhanced RS (SERS), confocal RS, optical-fiber RS, Fourier-transform RS, and laser-resonance RS improve the signal-to-noise ratio by enhancing either the signal intensity or signal collection (Kong et al., 2015).

### 2.2 Related Raman Spectroscopy Detection Techniques

Combining RS with other technologies extends its application under different conditions. For instance, SERS increases the Raman signal by approximately 10^5^–10^14^ times because of the molecules attached to the surface of the nanostructured metal, thereby extending the capacity to detect trace substances to single molecules (Wang et al., 2014; Aditi et al., 2015). When the effector molecule is adsorbed or located in the vicinity of the metal nanostructure, Raman scattering is enhanced due to the resonant interaction of light with the surface plasmons excited by the surface of the sample atom (electromagnetic enhancement). Chemical enhancement can be observed *via* the interaction between the molecules and electrons from the surface. Further advantages of SERS include accurate spectral width, detection of multiple labels under a single-wavelength laser, and no photo-bleaching (Fleischmann et al., 1974; Jeanmaire and Duyne, 1977). SERS activity of Ag^+^ staining was found to be slightly higher than that of Au nanoparticles (AuNPs) but significantly lower than that of Ag nanoparticles (AgNPs) (Athukorale et al., 2019). Therefore, SERS is a good option for researchers to obtain bands with higher intensities. Micro-RS is a combination of RS and microscopic analysis and is considered a powerful technique. RS and optical microscopy can be effectively combined using an excitation laser with wavelengths in the visible and near-infrared regions (Delhaye and Dhamelincourt, 2010). With features of being microscopic, *in situ*, multi-phase, stable, and having high spatial resolution, it can perform point by point scanning and obtain high-resolution three-dimensional images. However, changes in the Raman spectrum baseline may mask small differences in the Raman band, which are crucial for identification in a diagnostic model. Moreover, optical-fiber RS, the combination of RS and fiber-optic probes, provides an alternative to medical diagnosis of hollow organs. Fiber-optic probes used *in vivo* must address the signal-to-noise ratio as well as the redundant Raman signal generated by the laser-transmitting fiber itself. To avoid strong background signals in the fused silica fiber in the fingerprint area (600–1800 cm^–1^), some fiber probes have been introduced to high wavenumber regions (2400–3800 cm^–1^) (Pavel and Nicholas, 1900). In addition, RS, which was first applied in 1986, has evolved rapidly and can be used to collect signals several times to increase the signal-to-noise ratio. In addition, irradiation of a sample with a 1064 mm near-infrared laser provided by the Perkin-Elmer company greatly diminishes the fluorescent background and presents great potential for non-destructive structural analysis of chemical, biological, and biomedical samples (Nixon and Smith, 1986).

## 3 Application of Raman Spectroscopy for Oral Diseases

### 3.1 Dental Caries

Dental caries are characterized by demineralization of the inorganic portion and destruction of the organic substances of the teeth, including enamel, dentin, and cementum, leading to impairment of the teeth (Klokkevold, 2015). Cariogenic bacteria is a prerequisite for the occurrence of caries, which is closely related to the formation of dental biofilms on the surfaces of teeth. The organic acids produced by cariogenic bacteria result in enamel demineralization with loss of calcium and phosphates. The first indication of dental caries is white spots on the enamel caused by demineralization (Klokkevold, 2015).

Regarding the inorganic components in tooth tissue, the iconic bands are four internal vibration modes of ${\text{PO}}_{4}^{3-}$ at *ν*_1_ ~ 960 cm^−1^, *ν*_2_ ~ 430 cm^−1^, *ν*_3_ ~ 1043 cm^−1^, and *ν*_4_ ~ 590 cm^−1^, and of ${\text{CO}}_{3}^{2-}$ at ~1070 cm^–1^. The intensities of ${\text{PO}}_{4}^{3-}$ indicate mineralization degree, and numerous studies have adopted 960 cm^–1^ to check the demineralization. For instance, Al-Obaidi et al. constructed a Raman map based on 960 cm^–1^ intensity tooth Raman spectra for measuring the depth of the lesion based on the intensity change at 960 cm^–1^ (Al-Obaidi et al., 2019). Zhang et al. calculated the 960 cm^–1^ intensity of a carious tooth sample. Assuming that the mineral content at a normal site is 100%, they acquired the mineral content of lesions by measuring the ratio between the intensity of the lesion area and that of the normal area (*I*_lesion_/*I*_normal_) (Zhang et al., 2019). ${\text{CO}}_{3}^{2-}$ substituted ${\text{PO}}_{4}^{3-}$ in hydroxyapatite (HAP) is a more soluble phase presented in initially decayed enamel (Seredin et al., 2015).

Besides mineral content, crystallinity is another parameter indicating tooth damage. The full width at half maximum (FWHM) at ~960 cm^–1^ and ~1070 cm^–1^ is often used to estimate crystallinity. The narrower the peak width, the higher the mineral crystallinity. Suzuki et al. discovered that during the process of demineralization, the scattering peaks *ν*_1_ (960 cm^–1^), *ν*_2_ (430 cm^–1^), *ν*_3_ (1044 cm^–1^), and *ν*_4_ (591 cm^–1^) corresponding to ${\text{PO}}_{4}^{3-}$ are not shifted, while the peak width increases, indicating that the crystallinity of the enamel is impaired (Suzuki et al., 2019). Guentsch et al. calculated the FWHM of 960 cm^–1^ in an experimental biomimetic mineralization kit (BIMIN) group (12.2 cm^–1^), enamel group (12.5 cm^–1^), and dentin group (16.6 cm^–1^), and found increased crystallinity of caries-free teeth in the BIMIN group (Guentsch et al., 2019).

For dentin, the proportion of organic components is much higher than that in enamel, and the symbolic bands of the structural alteration of collagen are involved in estimating dentin caries. Intensities of ~1655 or ~1667 cm^–1^, ~1246 or ~1270 cm^–1^, and ~1450 cm^–1^ are assigned to amide I, amide III, and CH_2_, respectively, reflecting the structural information of collagen. In addition, the intensity ratio of amide I and phosphate *ν*_1_ (*I*_1650 cm−1_/*I*_960 cm−1_) was found to be related to the Knoop microhardness of tooth tissue, indicating that RS can be used to obtain the hardness of dentin caries as an alternative to invasive hardness testing (Alturki et al., 2020).

Previous studies have demonstrated that RS can be used for (1) detecting caries-related bacteria and early caries, (2) assessing the remineralization effect of drugs, (3) defining the margin of defective dentin, (4) exploring the effects of radiation therapy on tooth components, (5) evaluating new bonding systems, and (6) exploring the effects of quaternary ammonium salts (QAS) on cariogenic biofilms (Carvalho et al., 2013; Milly et al., 2014; Yang et al., 2014; Adachi et al., 2015; Seredin et al., 2015; Toledano et al., 2015a; Toledano et al., 2015b; Kerr et al., 2016; Pezzotti et al., 2017; Rodrigues et al., 2017; Occhi-Alexandre et al., 2018; Al-Obaidi et al., 2019; Daood et al., 2019; Guentsch et al., 2019; Hass et al., 2019; Lu et al., 2019; Miranda et al., 2019; Par et al., 2019; Suzuki et al., 2019; Toledano et al., 2019; Zhang et al., 2019; Alturki et al., 2020; Daood et al., 2020b; Gieroba et al., 2020) (**Table 1**).

**Table 1:** Summary of RS studies on dental caries.

Sample type	Target	Year	Authors	Raman shift		Meaning of the corresponding Raman shift
Detecting early caries, verifying demineralization models, and testing accuracy of other tools						
Dentin	Verifying intact and decayed dentin	2018	(Occhi-Alexandre et al., 2018)	1665 cm^–1^; 1453 cm^–1^; 1270 cm^–1^; 961 cm^–1^		Amide I; CH group; Amide III; Phosphate apatite
Enamel	Verifying demineralized enamel	2017	(Rodrigues et al., 2017)	~960 cm^–1^		Phosphate apatite
Teeth	Detecting caries	2014	(Yang et al., 2014)	–		–
Teeth	Verifying decayed teeth	2013	(Carvalho et al., 2013)	~575 cm^–1^; ~960 cm^–1^; ~1450 cm^–1^		Fluoridated apatite; Phosphate apatite; Organic matrix
Evaluating the remineralization of dental caries *in vitro*						
Dentin	Remineralization effect of zinc-containing amalgam restoration	2019	(Toledano et al., 2019)	–	Organic and inorganic components	
Enamel	Remineralization of carious enamel	2016	(Kerr et al., 2016)	960 cm^–1^	Phosphate apatite	
Dentin	Remineralization effect of self-etching zinc-doped adhesives	2015	(Toledano et al., 2015a)	–	Organic and inorganic components	
Dentin	Remineralization effect of zinc-containing amalgam loads	2015	(Toledano et al., 2015b)	–	Organic and inorganic components	
Enamel	Remineralization effect of PAA-BAG and BAG on WSL	2014	(Milly et al., 2014)	433 cm^–1^; 579 cm^–1^; 959 cm^–1^; 1043 cm^–1^	Phosphate apatite	
Distinguishing intact, infected, and affected dentin to define the margin of defective dentin precisely						
Dentin	Combining fluorescence spectra with Raman spectra	2012	(Almahdy et al., 2012)	960 cm ^–1^; 1340 cm ^–1^		Phosphate; Protein α-helices
Exploring the effects of radiation therapy on tooth components						
Dentin	Inorganic components	2019	(Campi et al., 2019)	590 cm^–1^; 1070 cm^–1^; 1267 cm^–1^		Fluoridated apatite; Phosphate apatite; Amide III
Teeth	Mineral composition; Collagen changes	2019	(Miranda et al., 2019)	1070 cm^–1^/960 cm^–1^; 1655 or 1667 cm^–1^/1246 or 1270 cm^–1^; 1655 or 1667 cm^–1^/1450 cm^–1^;		Carbonate/Mineral; Amide I/Amide III; Amide I/CH_2_
Teeth	Ratio of organic to inorganic components	2019	(Lu et al., 2019)	2931/960 cm^–1^		Protein/Mineral
Evaluating new bonding systems						
Adhesive	Ratio of uncured to cured unit	2019	(Par et al., 2019)	1639 or 1640 cm^–1^; 1609 or 1610 cm^–1^		Aliphatic C=C stretching; Aromatic C=C stretching
		2019	(Hass et al., 2019)			
Exploring the effect of quaternary ammonium salts (QAS) on cariogenic biofilms						
Biofilm	Effect of QAS on cariogenic biofilm changes	2020	(Daood et al., 2020b)	484 cm^–1^; 960 cm^–1^; 430 cm^–1^; 1070 cm^–1^		Polysaccharide; Phosphate; Carbonate
	
		2019	(Daood et al., 2019)
Biofilm	Metabolism of different biofilms	2020	(Gieroba et al., 2020)	–		–
Single cell of bacteria	Metabolic changes of single cell of bacteria after exposure to drugs	2020	(Tao et al., 2017)	2040–2300 cm^–1^		C–D vibration

WSL, white spot lesion; QAS, quaternary ammonium salts.

#### 3.1.1 Application of Raman Spectroscopy for Detecting Caries-Related Bacteria and Early Caries

In dental plaque, the cariogenic bacteria are wrapped in an organic matrix of polysaccharides, proteins, and DNA, which enhances resistance to host defense and antimicrobial agents (Selwitz et al., 2007). Endogenous cariogenic bacteria (mainly *Streptococcus mutans*, *Streptococcus sobrinus*, and *Lactobacillus* spp) ferment carbohydrates and produce organic acids, resulting in local pHs below the critical value and demineralization of teeth (Featherstone, 2004; Selwitz et al., 2007).

RS can detect metabolic differences to facilitate the differentiation between biofilms. Gieroba et al. collected and analyzed Raman spectra from single biofilms of *S. mutans* CAPM 6067, *Streptococcus sanguis* ATCC 10556, and several serotypes of *S. sobrinus*. The major differences were concentrated in the region representing lipids, amides, and carbohydrates, reflecting the corresponding biological characteristics. For example, the highest and lowest amide bands in several biofilms were different, indicating different protein compositions of the biofilms and adhesive and cariogenic characteristics (Gieroba et al., 2020). Daood et al. used the changes in Raman spectra to assist in evaluating the effect of quaternary ammonium on cariogenic biofilms. With exposure to QAS, the intensity of 484 cm^–1^ in the Raman spectra, representing polysaccharides or carbohydrates, was significantly reduced, and the change was in concentration-dependent and time-dependent patterns (Daood et al., 2020a). Tao et al. focused on heavy water (D_2_O) based on single-cell Raman microspectroscopy (D_2_O-Raman) and analyzed the Raman spectral region from 2040 to 2300 cm^–1^, representing the C–D vibration band, to evaluate the metabolic status of the *S. mutans* UA159, *Streptococcus gordonii* ATCC10558, *S. sanguinis* ATCC10556, and *Lactobacillus fermentum* ATCC9338 oral bacteria after drug exposure. They distinguished antibiotic-sensitive and -resistant *S. mutans* (Tao et al., 2017).

The DIAGNOdent pen (Germany) is a laser pen that can detect caries *in vivo* by collecting fluorescence. To evaluate the laser pen detection capability, RS has been applied to test changes in carious teeth, and Rodrigues et al. established an enamel demineralization model *in vitro* with cattle tooth blocks and chose phosphate apatite peaks at ~960 cm^–1^ to estimate demineralization (Rodrigues et al., 2017). Carvalho et al. focused on the changes in fluoridated apatite, phosphate apatite, and organic matrix in carious teeth, which present in Raman spectra as ~575 cm^–1^, ~960 cm^–1^, and ~1450 cm^–1^, respectively. As demineralization progressed, the intensities of ~575 cm^–1^ and ~960 cm^–1^ declined significantly and were negatively correlated with the fluorescence detected by the DIAGNOdent pen. This proved that fluoridated apatite and phosphate apatite decreased in caries and verified the caries detection accuracy of the DIAGNOdent pen (Carvalho et al., 2013). Point-scan and wide-field Raman imaging have also been investigated for caries detection as well as application in laser pens (Yang et al., 2014). RS combined with two-dimensional (2D) charge-coupled-device cameras can be assembled into wide-field Raman imaging, which is faster for diagnosing dental caries. Additionally, to compare the penetration depth of a photosensitizer (erythrosine) on intact dentin and decayed dentin *in vitro*, RS was applied to verify the intact and decayed dentin by providing organic and inorganic information (Occhi-Alexandre et al., 2018). However, the results were mainly obtained from tooth slices *in vitro*, and to determine whether saliva and bacteria interfere with the process *in vivo* requires further investigation. In addition, Almahdy et al. collected Raman and fluorescence spectra of carious tooth tissues (Almahdy et al., 2012). Combined with three fluorescence signals (porphyrin fluorescence, putative infected dentin signal, and affected dentin signal), two Raman signals, phosphate at 960 cm^–1^ and protein at 1340 cm^–1^, enabled differentiation between intact, infected, and affected dentin, indicating that vital transformations of phosphate and protein α-helices are presented. More information could be obtained by investigating organic-related bands more intensively.

#### 3.1.2 Raman Spectroscopy to Evaluate the Remineralization of Dental Caries *In Vitro*

Enamel is the most superficial tissue of teeth and is covered by dental plaque, which consists mainly of bacteria. When sugar and other fermentable carbohydrates reach the bacteria and produce acids, teeth demineralization begins. Conversely, when sugar consumption ceases, saliva washes away the sugars and buffers the acids. Calcium and phosphates then enter the teeth again, resulting in remineralization. Thus, a cavity occurs if demineralization overtakes remineralization over time (Klokkevold, 2015). Kerr et al. evaluated the remineralization status of carious enamel treated with high-frequency microwave energy to sterilize and adjust pH by estimating the 960 cm^–1^ intensity change (Kerr et al., 2016). For promoting enamel white spot lesion (WSL) remineralization, Raman spectra were also employed to assess the potential of bioactive glass (BAG) powder and BAG containing polyacrylic acid (PAA-BAG) (Milly et al., 2014). Four internal vibration modes of ${\text{PO}}_{4}^{3-}$ at 433 cm^–1^, 579 cm^–1^, 959 cm^–1^, and 1043 cm^–1^ were adopted to estimate enamel demineralization and remineralization. The intensity at 959 cm^–1^ was the strongest among the four peaks.

Regarding remineralization in dentin, it is worth mentioning that Toledano et al. adopted many iconic bands to evaluate dentin changes from relative mineral concentration, crystallinity, and organic composition of dentin. Thus, the positive effects of zinc-containing amalgam mechanical loads, self-etching zinc-doped adhesives, and zinc-containing amalgam restoration on dentin remineralization before and after 24 h and after 3 weeks were clarified (Toledano et al., 2015a; Toledano et al., 2015b; Toledano et al., 2019).

### 3.2 Periodontal Diseases

Periodontitis is a chronic inflammatory disease of tooth-supporting tissues caused by pathogenic bacterial species located in the subgingival niche. Periodontal pathogens often cause the destruction of periodontal tissues, mainly by expressing toxic factors and triggering an inflammatory host response. According to the “Keystone-Pathogen Hypothesis” (2012) (Hajishengallis et al., 2012) and polymicrobial synergy and dysbiosis model (2012) (Hajishengallis and Lamont, 2012), *Porphyromonas gingivalis* is a key periodontal pathogen, the toxic factors of which, including fimbriae, capsule, and gingipain, can destroy periodontal tissues directly and trigger an inflammatory response. In addition, it can trigger the dysbiosis of subgingival flora and finally transform into a pathogenic biofilm with higher virulence-related gene expression and stronger destructive inflammation (Curtis et al., 2020). Previous studies have reported that RS can be used for (1) detecting subgingival bacteria, (2) analyzing changes in saliva, and (3) depicting bone transformation (**Table 2**).

**Table 2:** Summary of RS studies on periodontal disease.

Sample type	Target	Year	Authors	Raman shift	Meaning of the corresponding Raman shift
Assisting diagnosis and detection of inflammatory factors and composition changes					
Periodontal ligament	Protein secondary structure	2020	(Perillo et al., 2020)	1307 cm^−1^; 1230–1250 cm^−1^; 1240–1270 cm^−1^; 1620 cm^−1^; 1668 cm^−1^; 1680 cm^−1^;2930 cm^−1^; 2875 cm^−1^; 2970 cm^−1^	α-helix; β-sheet; random coil; β-sheet or collagen 3_10_-helix, β-turn and β-sheet secondary structure; CH_3_ and CH_2_
Saliva	IL-1β; TNF-α	2020	(Yang et al., 2020)	1335 cm^–1^; 1590 cm^–1^	DTNB; 4-MBA
Saliva	Sialic acid	2019	(Hernández-Cedillo et al., 2019)	1002 cm^–1^, 1237 cm^–1^, and 1391 cm^–1^; OR 910 cm^–1^, 1171 cm^–1^, and 1360 cm^–1^	Sialic acid
GCF	Mineral–matrix ratio; Carbonate apatite–hydroxyapatite ratio;	2014	(Jung et al., 2014)	984 cm^−1^/1667 cm^−1^; 1088 cm^−1^/984 cm^−1^;	Phosphate/Amide I; Carbonate/Phosphate;
Saliva	Carotenoids	2011	(Gonchukov et al., 2011)	1155 cm^–1^; 1525 cm^–1^	C–C; C=C
Detecting metabolites of periodontal bacteria					
*P. gingivalis* ATCC 33277	–	2016	(Pezzotti et al., 2016)	–	–
*Pr. nigrescens* ATCC 25261; *Pr. intermedia* ATCC 25611; *P. gingivalis* W50; Fe(III)PPIX.OH; [Fe(III)PPIX]_2_O	Heme pigment	2003	(Smalley et al., 2003)	338 cm^–1^; 370 cm^–1^; 1549 cm^–1^; 1570 cm^–1^; 1580 cm^–1^; 1618 cm^–1^; 1621 cm^–1^	–
Distinguish subgingival bacteria					
*P. gingivalis; A. actinomycetemcomitans; Streptococcus* spp.	–	2021	(Witkowska et al., 2021)	–	–
*F. nucleatum,; S. mutans; V. dispar; A. naeslundii; Pr. nigrescens*	–	2020	(Kriem et al., 2020)	–	–

#### 3.2.1 Raman Spectroscopy to Assist in Detecting Subgingival Bacteria and Analyzing Changes of Saliva

Pioneering research began in 1999 when RS was used to detect the metabolites of periodontal bacteria. Evidence was found that an increase in spectral intensity at 1002 cm^–1^ with time implies that the heme pigment is gradually accumulated on the cell surface when *P. gingivalis* is incubated on a blood plate. Moreover, *P. gingivalis* can synthesize the iron trivalent oxidation state Fe(III)PPIX with a band at 1373 cm^–1^ on a horse blood plate while synthesizing the iron divalent oxidation state Fe(II)PPIX with a band at 1359 cm^–1^ without horse blood. In 2003, researchers applied Mohs, Raman, and UV–vis spectrophotometry to characterize the heme pigment of *Prevotella nigrescens* ATCC 25261 and *Prevotella intermedia* ATCC 25611 (Smalley et al., 2003). They also explored the changes in heme pigment under different pH conditions. In 2016, *in situ* Raman microprobe spectroscopy was used to track the metabolic changes of *P. gingivalis* on the polished surfaces of bioceramics of the antibacterial substance silicon nitride (Si_3_N_4_), revealing the formation of peroxynitrite in *P. gingivalis* (Pezzotti et al., 2016).

In addition, Raman spectra can be used to distinguish different subgingival bacteria using data analysis. In 2020, Kriem et al. distinguished *Fusobacterium nucleatum, S. mutans, Veillonella dispar, Actinomyces naeslundii*, and *Prevotella nigrescens* with 100% accuracy in planktonic state. The accuracy of distinguishing *S. mutans*, *V. dispar*, and *A. naeslundii* single-species biofilms was 76%, and that for the others was 90% or higher (Kriem et al., 2020). Based on advanced technologies, in 2021, Witkowska et al. designed a standard Raman spectral detection process to differentiate different serotypes of *P. gingivalis, Aggregatibacter actinomycetemcomitans*, and *Streptococcus* spp.

The detection process applied microfluidics, Fe_2_O_3_@AgNPs combined with Ag/Si substrates, and successfully distinguished *P. gingivalis* from *A. actinomycetemcomitans* and *Streptococcus* spp. by principal component analysis (PCA) with an accuracy of 82–91%. They also confirmed the effectiveness of this detection system in a saliva environment (Witkowska et al., 2021). The detection process provides a feasible method for detecting periodontal pathogens in a clinical environment.

Regarding diagnosis, saliva and GCF have always been prominent as bodily fluids that can be obtained noninvasively and contain effective information as well as interfering noise. One important research approach for analyzing Raman spectra containing a large amount of information is to focus on peaks representing target substances, such as carotene, carotenoids, and sialic acid (SA).

In 2011, Gonchukov et al. collected saliva from 10 patients with periodontitis and 10 healthy subjects (Gonchukov et al., 2011). There were unique peaks in the periodontitis group at 1155 and 1525 cm^–1^, representing C–C and C=C, respectively (Darvin et al., 2010), which indicates the existence of carotenoids (Darvin et al., 2009) and proves that as the severity of the periodontal disease increases, the total antioxidant level in saliva also rises (Kim et al., 2010). In 2014, Camerlingo et al. compared the Raman spectra of GCF collected from healthy and periodontitis patients and found that the band at 1537 cm^–1^, which represents the isomerization product of the C=C group related to the degraded carotene, only appeared in the latter group (Camerlingo et al., 2014). The aforementoined studies confirm that carotenoid concentration may be beneficial in the diagnosis of periodontitis. Furthermore, SA is present in several proteins related to periodontitis (Ide et al., 2003). Hernandez-Cedillo et al. reported the potential of SA in the auxiliary diagnosis of periodontal diseases. In 2019, they collected saliva samples from patients with periodontitis or gingivitis as well as healthy controls. The peaks at 1002 cm^–1^,1237 cm^–1^, and 1391 cm^–1^ or 910 cm^–1^, 1171 cm^–1^, and 1360 cm^–1^ were compared with the standard SA peaks, indicating that the concentrations of SA in each group were significantly different (Hernández-Cedillo et al., 2019). Interleukin-1β (IL-1β) and tumor necrosis factor-α (TNF-α) are important cytokines in periodontitis, and their concentrations in GCF are higher with periodontitis (Klokkevold, 2015). Yang et al. labeled IL-1β and TNF-α with 5,5’-Dithiobis-(2-nitrobenzoic acid) (DTNB) and 4-mercaptobenzoic acid (4-MBA), respectively, and then collected the salivary surface-enhanced Raman spectra of patients with different periodontal conditions (Yang et al., 2020). The relative area values of the IL-1β and TNF-α peaks at 1335 cm^–1^ and 1590 cm^–1^, respectively, were calculated as the Raman intensity corresponding to the concentration of inflammatory factors, and significant differences were found between the three groups. The spectral characteristics of saliva reflect metabolic changes in periodontal tissues, and are of vital importance in the diagnosis of periodontitis. However, the composition of saliva is affected by many factors, such as diet and physical condition. Thus, a multi-center and large-sample study is necessary to acquire reliable spectral characteristics of different periodontal conditions.

Regarding Raman spectra of GCF, Jung et al. studied the changes in GCF composition during orthodontic tooth movement with RS. RS showed the degree of bone mineralization and accumulation of carbonate in the apatite lattice. During the alveolar bone remodeling, the mineral–matrix ratio decreased and the carbonate apatite–hydroxyapatite ratio increased. It is speculated that this results from insufficient mineralization during alveolar bone remodeling (Jung et al., 2014). Perillo et al. observed the changes in vibrational modes of proteins (amide I and amide III bands) and CH_2_ and CH_3_ modes in the periodontal ligament 2, 7, and 14 days after adding orthodontic force to obtain the molecular arrangements and conformational changes. The Raman spectra of the α-helix, 3_10_-helix, β-turn, β-sheet and random coil in the amide I and amide III bands representing the secondary structure of the protein changed markedly with orthodontic tooth movement. The α-helical and the intensity of the entire amide I band were reduced compared with the control periodontal ligament sample. Compared to the α-helical mode, the remaining component mode of amide I became wider and stronger. The information from the Raman spectra provided quantitative insight into when and how the periodontal ligament molecular arrangement changed (Perillo et al., 2020).

#### 3.2.2 Raman Spectroscopy to Depict Bone Transformation

In addition to saliva, GCF, and gingival tissue, periodontal hard tissue has also been considered for possible applications of RS. In 2019, Gatin et al. reported the application of RS to evaluate the bone differences between a periodontitis patient and a healthy patient before and after maxillary sinus lift surgery. Octacalcium phosphate (OCP) and amorphous HAP presented obvious peaks at 955–960 cm^–1^, representing immature bone. Moreover, HAP crystals and biological HAP-based bone substitutes showed bands at 960–965 cm^–1^, representing mature bone. Based on this analysis, t bone samples before surgery and 8 months after healing revealed that the two patients’ samples had peaks at 955–960 cm^–1^ before surgery, and only peaks at 960–965 cm^–1^ presented after surgery. The broad fluorescence peaks appearing at 800–900 cm^–1^ represented collagen, and the changes in specific peaks could be used to quantify the healing process (Gatin et al., 2019). Raman spectra clearly depict the transformation from immature to mature bone.

### 3.3 Oral Cancer

Oral cancers are cancerous growths in the mouth, and they are life-threatening if not diagnosed and treated early. The most common type of oral cancer is oral squamous cell carcinoma (OSCC) (Klokkevold, 2015), and biopsy is the gold standard for OSCC diagnosis. Doctors can estimate the severity of the condition according to the International Union Against Cancer’s primary tumor, regional lymph nodes, and metastasis (TNM) classification and the World Health Organization (WHO) histologic grading system. To date, RS has mainly been explored for the diagnosis and classification of oral cancer. A few studies have also collected data and studied the application of RS in the treatment and prognosis of oral cancer. **Table 3** summarizes the literature on RS for oral cancer.

**Table 3:** Summary of RS studies on oral cancer.

Sample type	Sample numbers	Year	Authors	RS (spectral region; laser used)	Data analysis methodology
Clarifying tumor stage, histological classification and cancer subtype					
Tumor resection specimen	OSCC = 20; VC = 4; OLK = 5; N = 8	2019	(Madathil et al., 2019)	SERS; 500–1800 cm^–1^; 488 nm	PCA-DA
Serum	Buccal cancer = 40; Tongue cancer = 50; Floor of mouth cancer = 45	2018	(Xue et al., 2018)	SERS; 200–1800 cm^–1^; 633 nm	PCA-LDA; LOOCV
Tissue engineering models	Models = 27: N (NOF; NOK); Dysplastic (DOK; D19; D20); HNC (Cal27; SCC4; FaDu)	2017	(Mian et al., 2017)	600–1800 cm^–1^ and 2800–3400 cm^–1^; 532 nm	PCA-LDA; CA
Buccal pouch tissue	*Ex vivo* = 115; *In vivo*; sequential = 60; *In vivo* follow-up = 6	2015	(Kumar et al., 2016)	1200–1800 cm^–1^;785 nm	PCA; PCA-LDA
Cells	Radioresistant cell sublines (70Gy-UPCI : SCC029B; 50Gy-UPCI : SCC029B); Parental oral cancer cell lines (UPCI : SCC029B)	2014	(Yasser et al., 2014)	900–1800 cm^–1^; 785 nm	PCA
Differentiating diagnosis between normal, precancerous lesions and cancer					
Tumor resection specimen	OSCC = 20; VC = 4; OLK = 5; N = 8	2019	(Madathil et al., 2019)	SERS (catheter (5–6 µm)); 500–1800 cm^–1^; 488 nm	PCA-DA
Tissue engineering models	Models = 27: N (NOF; NOK); Dysplastic cells (DOK; D19; D20); HNC (Cal27; SCC4; FaDu)	2017	(Mian et al., 2017)	600–1800 cm^–1^ and 2800–3400 cm^–1^	PCA-LDA; CA
*In vivo*	OSCC = 113; OSMF = 25; OLK = 33; HV = 28	2014	(Krishna et al., 2014)	900–1750 cm^–1^; 785 nm	PCA-LDA
Differentiating diagnosis between different tumors (OSCC, other oral tumors and carcinomas in other systems)					
Serum	HV = 39; Breast cancer = 42; Colorectal cancer = 109; Lung cancer = 33; Oral cancer = 17; Ovarian cancer = 13	2019	(Moisoiu et al., 2019)	SERS; 600–1800 cm^–1^; 532 nm	PCA-LDA
Tissue frozen section	OSCC = 20; VC = 4; OLK = 5; N = 8	2019	(Madathil et al., 2019)	SERS(catheter (5–6 µm)); 500–1800 cm^–1^; 488 nm	PCA-DA
Exfoliated cell	N = 13; OLK = 13; OSCC = 10	2019	(Ghosh et al., 2019)	200–2000 cm^–1^; 785 nm	PCA-LDA; k-fold cross-validation
Exfoliated cell	Tumor = 16; Contralateral mucosa = 16; HT = 20	2019	(Sahu et al., 2019)	800–1800 cm^–1^; 785 nm	PCA-LDA; LOOCV
Exfoliated cell	HV = 20; TH = 20; OPL = 27	2017	(Sahu et al., 2017)	800–1800 cm^–1^; 785 nm	PCA-LDA
Serum	OSCC = 135; MEC = 90; HV = 145	2017	(Tan et al., 2017)	SERS; Fingerprint regions (200–1800 cm^–1^); 633 nm	PCA-LDA
Saliva and exfoliated cell	Person: HV = 18; OSCC = 18; Spectra: Saliva = 180; Cell = 120	2016	(Connolly et al., 2016)	SERS; 800–1800 cm^–1^; 785 nm	PCA-LDA; PCA-LR
Serum	PA = 20; WT = 21; MEC = 19; HV = 31	2015	(Yan et al., 2015)	SERS; 200–1800 cm^–1^; 633 nm	SVM
Tissue section	HV = 20; PA = 20; WT = 20	2011	(Yan et al., 2011)	800–1800 cm^–1^; 785 nm	SVM
Obtaining more important information to elevate differentiation efficacy					
Exfoliated cell	N = 13; OLK = 13; OSCC = 10;	2019	(Ghosh et al., 2019)	200–2000 cm^–1^; 785 nm	PCA-LDA; k-fold cross-validation
Cells	Nucleolus, nucleus and cytoplasm: SCC-4 = 60; DOK = 60; N = 60	2017	(Carvalho et al., 2017)	2800–3600 cm^–1^; 532 nm	PCA-FDA
Dehydrated cancer cell	–	2013	(Panikkanvalappil et al., 2013)	SERS; 400–2000 cm^–1^; 532 nm	–
Selecting better analysis methods to elevate differentiation efficacy					
Tissue section	N = 36; Tumor = 44 (tongue, buccal mucosa, gingiva)	2019	(Jeng et al., 2019)	700–2000 cm^–1^; 532 nm	PCA-LDA; PCA-QDA; LOOCV; k-fold cross-validation
Tumor resection specimen	OSCC = 14; N = 11	2016	(Cals et al., 2016)	400–1800 cm^–1^; 785 nm	PCA-(h)LDA
Focusing on biomarkers to elevate differentiation efficacy					
Saliva	OSCC = 6; HV = 5	2020	(Fălămaş et al., 2020)	SERS; 600–1720 cm^–1^; 785 nm	PCA
Saliva	OSCC = 3; Lymphoma = 1; Actinomyces infection = 1; HV = 3	2020	(Falamas et al.)	100–3200 cm^–1^; 785 nm	PCA-LDA
Saliva	Oral dysplasia = 10; HV = 10	2020	(Daniel et al., 2020)	SERS; -	–
Saliva	OSCC = 3; HV = 3	2019	(Han et al., 2019)	SERS; 1100–1700 cm^–1^; 638 nm	Mann–Whitney U-test
Tissue section	OSCC = 13; N = 11	2016	(Chen et al., 2016)	800–1800 cm^–1^; 488 nm	Multivariate curve resolution with alternating least squares
Assessing surgical margins					
Tissue resection specimen	OSCC (mandibular bone) = 20	2018	(Barroso et al., 2018)	2500–4000 cm^–1^; 671 nm	PCA-LDA; Mann–Whitney U-test; ROC
Tumor resection specimen	OSCC (tongue) = 20	2016	(Barroso et al., 2016)	2500–4000 cm^–1^; 671 nm	Mann–Whitney U-test
Tumor resection specimen	OSCC = 14; N =11	2016	(Cals et al., 2016)	400–1800 cm^–1^; 785 nm	PCA-(h)LDA
Tumor resection specimen	OSCC (tongue) = 14	2015	(Barroso et al., 2015)	2500–4000 cm^–1^; 671 nm	Mann–Whitney U-test; ROC
Predicting oral cancer recurrence					
Exfoliated cell	Tumor = 16; Contralateral mucosa = 16; HT = 20	2019	(Sahu et al., 2019)	800–1800 cm^–1^; 785 nm	PCA-LDA; LOOCV
*In vivo*	Tumor and contralateral normal mucosa = 99	2017	(Malik et al., 2017)	785 nm	PCA-LDA; LOOCV
Serum	Recurrence = 10; No-recurrence = 12	2015	(Sahu et al., 2015)	700–1800 cm^–1^; 785 nm	PCA-LDA; LOOCV

OSCC, oral squamous cell carcinoma; N, normal; HV, healthy volunteers; TH, tobacco habit; OPL, oral premalignant conditions; HNC, head and neck cancer; VC, verrucous carcinoma; OSMF, oral submucous fibrosis; OLK, leukoplakia; MEC, mucoepidermoid carcinoma; PA, pleomorphic adenoma; WT, Warthin’s tumor.

#### 3.3.1 Diagnosis and Classification of Oral Cancer

In 2004, Krishna et al. first studied the potential of RS for detecting oral cancer (Krishna et al., 2004). They collected Raman spectra from healthy and malignant epithelial cells and differentiated them using PCA. Subsequently, many studies have validated this conclusion. In summary, Raman spectra have been studied to (1) clarify the tumor stage and histological classification of OSCCs, (2) distinguish OSCCs from precancerous lesions and other cancers, and (3) improve the accuracy of diagnostic models.

Clarifying the tumor stage and histological classification is critical for evaluating patients’ conditions and choosing the best treatment plan. Xue et al. established a diagnostic model based on the spectra of serum samples from 135 patients with OSCCs using PCA with linear discriminant analysis (PCA-LDA). The total accuracies of the diagnostic model in identifying tumors at different stages, distinguishing lymph node involvement, and distinguishing between different histological grades were 90.4%, 85.9%, and 90.4%, respectively (Xue et al., 2018). A novel SERS catheter (5–6 µm) helped to successfully obtain and differentiate healthy cells, moderate OSCCs, and severe OSCCs with an accuracy of 97.84% (Madathil et al., 2019). Surprisingly, even subtypes of head and neck cancer cells could be identified by analyzing the spectra from tissue engineering models. Mian et al. successfully identified subtypes of head and neck cancer cells (Mian et al., 2017). Significant differences in the spectra were observed in the lipid content (2881 cm^–1^) and protein structure (amides I and III), the peaks of which are associated with several amino acids and nucleic acids (600 cm^–1^ to 1003 cm^–1^). Therefore, doctors can choose chemotherapy or radiotherapy based on the known subtypes of cancer. In addition, Yasser et al. successfully distinguished radioresistant cell sublines (70Gy-UPCI : SCC029B; 50Gy-UPCI : SCC029B) from parental oral cancer cell lines (UPCI : SCC029B) (Yasser et al., 2014). PCA presented a minor overlap between three clusters, indicating a large difference between three cell lines. Furthermore, Kumar et al. explored the change in differentiation efficacy during the cancer-inducing process, and the accuracy of cancer identification increased during the first 7 weeks, remained steady from 8 to 11 weeks, and exceeded 80% by the 14th week (Kumar et al., 2016).

Furthermore, RS has been found to be a powerful tool for distinguishing OSCCs from precancerous lesions. Krishna et al. studied the potential of Raman spectra obtained *in vivo* directly to differentiate malignant lesions (OSCCs, oral submucous fibrosis (OSMF) and leukoplakia (OLK)) in the oral cavity. The accuracy was 85% in HV, 89% for OSCCs, 85% for OSMF, and 82% for OLK. For spectra classified as normal and abnormal, the sensitivity and specificity were 94.2% and 94.4%, respectively (Krishna et al., 2014). However, another study reported that OSMF, OLK, and lichen planus were highly misclassified as OSCCs or habitues without lesions in the Raman spectra of sera (Dumal et al., 2020). OSCCs, verrucous carcinomas, and OLK were differentiated with 97.24% accuracy by taking thin cryosections of tissue specimens in a novel SERS catheter (5–6 µm) (Madathil et al., 2019). Moreover, RS combined with cytopathology can distinguish oral precancerous lesions (OPLs), healthy tobacco users (HT), and HV (Ghosh et al., 2019). The sensitivity of the OPL group was identified as approximately 77% when analyzed spectrally, which was higher than patient-wise, with a sensitivity of approximately 70%. The main changes in the spectra of OPLs were related to nucleic acids and proteins. This is consistent with changes in protein and DNA corresponding to cellular physiological changes during poor cell proliferation (Sahu et al., 2017). Connolly et al. obtained unlabeled spectra from saliva and exfoliated cells of HV and OSCC patients using SERS and established diagnostic models using PCA-LDA and PCA with logistic regression (PCA-LR) diagnostic algorithms. Consequently, it was concluded that the saliva and exfoliated cells could identify HV and patients with OSCCs, with sensitivities of 89% and 73% and overall accuracies of 68% and 60%, respectively (Connolly et al., 2016). In brief, the accuracy of Raman spectra from exfoliated cells in differentiating diagnoses must be improved. Misclassification of Raman exfoliative cytology also indicated field cancerization changes. The higher the misclassification rate between spectra of contralateral normal tissue and tumor tissue, the more similar the exfoliated cells are (Sahu et al., 2019).

Researchers have differentiated OSCCs, other oral cancers (such as verrucous carcinoma, mucoepidermoid carcinoma, parotid pleomorphic adenoma, and Warthin’s tumor) (Yan et al., 2011; Yan et al., 2015; Tan et al., 2017; Madathil et al., 2019), and cancers in other regions (such as breast, colorectal, lung, and ovarian) (Moisoiu et al., 2019). The difference in the SERS spectra of sera between OSCCs, mucoepidermoid carcinomas, and healthy humans is mainly represented by nucleic acids and proteins. The spectral differences are mainly distributed in the spectral bands represented by the specific molecular structures of carotenoids and lipids. OSCCs were successfully distinguished from a control group with a sensitivity of 80.7% and specificity of 84.1% (Tan et al., 2017). Another study has also attempted to distinguish OSCCs from parotid pleomorphic adenomas, Warthin’s tumors, and mucoepidermoid carcinomas by support vector machine (SVM) according to the SERS information of sera. The results showed that the SVM had a favorable effect on SERS spectral classification, with an accuracy of 84.1–88.3%, sensitivity of 82.2–97.4%, and specificity of 73.7–86.7%. Although this method can easily differentiate mucoepidermoid carcinoma from the other two benign tumors, it is difficult to distinguish between the two benign tumors themselves (Yan et al., 2011; Yan et al., 2015). Moisoiu et al. successfully discriminated several cancers with an accuracy of 88% for oral cancer, 76% for breast cancer, 86% for colorectal cancer, 59% for lung cancer, and 80% for ovarian cancer (Moisoiu et al., 2019). AgNP substrate enhanced the signal in serum samples and PCA-LDA differentiated spectra in different groups.

Obtaining more important information can increase the differentiation efficacy. Ghosh et al. combined Fourier-transform infrared spectroscopy (FTIR) and RS to increase the classification accuracy from 85% (FTIR) and 82% (RS) to 98% (Ghosh et al., 2019). Spectra of DNA from dehydrated cancer cells and high-wavenumber regions of spectra have attracted the attention of researchers. Panikkanvalappil et al. provided a new method for improving the accuracy of the diagnostic model. Their study found that the conformational induction of DNA from dehydrated cancer cells presents a series of unique Raman-labeled bands. According to these bands, cancer and healthy cell DNA can be distinguished. It is speculated that nucleobase damage in tumor cell DNA and subsequent changes in the electron cloud during the dehydration-driven conformational change results in a Raman spectral change (Panikkanvalappil et al., 2013). The high-wavenumber region contains more distinct information for identifying subcellular structure, i.e., the nucleus and cytoplasm, than the fingerprint region. Carvalho et al. analyzed the spectra of the nucleolus, nucleus, and cytoplasm of oral epithelial carcinomas (SCC-4), dysplasia (DOK) cell lines, and normal oral epithelial cells. The O–H bond from cell-membrane-bound water or intracellular fluid provided key information for distinguishing cell lines. The sensitivity and specificity of cytoplasm recognition were up to ~100% and 97%, respectively. Regarding nuclear recognition, the specificity was 99%. Compared to other methods, high-wavenumber regions provide more information about subcellular structures to distinguish between normal, premalignant, and malignant tissues (Carvalho et al., 2015; Carvalho et al., 2017).

In addition, improving the efficiency of the classifier can increase the distinguishing accuracy. Jeng et al. gathered the spectra from 36 normal and 44 OSCC tissues and analyzed them with PCA, followed by LDA or quantitative discrimination analysis (QDA). The accuracies of the PCA-LDA and PCA with QDA (PCA-QDA) classifiers were 81.25% (sensitivity: 77.27%, specificity: 86.11%) and 87.5% (sensitivity: 90.90%, specificity: 83.33%), respectively. PCA-QDA showed better classification efficiency than PCA-LDA (Jeng et al., 2019). Moreover, Cals et al. adopted PCA with (hierarchical) LDA (PCA-(h)LDA) (Cals et al., 2016). Because there is a large difference between lipid, nerve, and tumor tissue, researchers first differentiated lipids and nerves from other tissues and then distinguished OSCCs from the remaining components (squamous epithelium and connective tissue, muscle, and glands). Compared to the one-step PCA-LDA model, the two-step PCA-(h)LDA model presented higher accuracy (91% accuracy; sensitivity: 100%, specificity: 78%). However, the PCA-(h)LDA model also increased the data analysis workload.

Furthermore, focusing on biomarkers is also a way to distinguish between normal and abnormal tissues. Chen et al. compared Raman spectra decomposed by multivariate curve resolution with alternating least squares (MCR-ALS) with the standard Raman spectra of keratin, a well-known molecular marker of OSCC. However, some spectra were neither divided into the OSCC group nor the normal group, and as a result, with different classification methods of suspicious samples, the sensitivity differed widely (77% or 92%) (Chen et al., 2016). Recently, Fălămaş et al. attempted to differentiate six OSCC patients from five HV by salivary Raman spectra (Fălămaş et al., 2020). They noticed that thiocyanate is not only an indicator of smokers (Tsuge et al., 2000) but also related to cancer (Shiue, 2015). The characteristic band of thiocyanate is 2126 cm^–1^, the intensity of which was higher in the cancer group than in the healthy group. The peak at 738 cm^–1^ is another characteristic band of thiocyanate, contributing to the differentiation of the two groups by PCA. Fălămaş et al. also found that the peaks at 752, 884, 928, 989, and 1047 cm^–1^, representing tryptophan, collagen, proline, and glycogen, respectively, contributed to finding the biggest difference between OSCC patients and HV in salivary spectra (Falamas et al.). Daniel et al. also concentrated on the presence of thiocyanate at 2108 cm^–1^ in the SERS spectra of saliva among smokers (Daniel et al., 2020). However, due to the small sample size, whether thiocyanate can be a potent biomarker for recognizing cancer patients requires further exploration. S100 calcium-binding protein P (S100P) mRNA has been reported as a valid salivary biomarker for oral cancer detection without periodontitis interference (Y.-S. et al., 2017). Han et al. designed a sandwich assay format consisting of oligonucleotides, AuNPs as the SERS substrate, and malachite green isothiocyanate as a reporter molecule to quantify S100P mRNA in saliva (Han et al., 2019). The concentration of S100P mRNA was three times higher in the oral cancer group than in the healthy group.

#### 3.3.2 Treatment and Prognosis of Oral Cancer

The extent to which the cancerous tissue can be removed by a clinician (i.e., whether it can be completely removed or not) significantly affects the prognosis. It has been reported that the 5-year disease-free survival rate significantly declines in patients with inadequate surgical margins. Even after surgical treatment and radiotherapy or chemotherapy, malignant lesions may still recur, which directly affects the prognosis and patients’ quality of life. Thus, assessing surgical margins and the potential for oral cancer recurrence are of major importance. RS shows the application potential and unique advantages of these two aspects.

For instance, frozen section is an intraoperative choice to assess whether the surgical margin is adequate. This technique works well for soft tissues, but it is difficult to use it to assess bone edges (Nieberler et al., 2016). RS has the potential to distinguish between malignant and normal tissues. The Raman map composed of the spectra of all sites of a specimen is a candidate method for determining the surgical margins more accurately during operation. Barroso et al. found that the main factor in distinguishing a tumor from surrounding healthy tissue is water concentration (Barroso et al., 2015). Based on this, they constructed a 2D Raman map to observe the change in water concentration from tumor tissue to the surrounding healthy tissue. In 2016, Barroso et al. (Barroso et al., 2016) applied the ratio of 3390 cm^–1^ (O–H stretching band of water) to 2935 cm^–1^ (C–H stretching band of lipids and proteins) as an indicator of water concentration and then assigned different colors to various water concentrations for 2D Raman map construction. In the 4–6 mm transition region between the tumor and surrounding normal tissue, the water content in the tissue ranged from 76% ± 8% in the tumor tissue to 54% ± 24% in the surrounding healthy tissue. The 2D Raman map presented the water concentration transformation from the tumor tissue to the surrounding healthy tissue directly, providing valid information for clinicians to determine the surgical edge more accurately. Two years later, Barroso et al. further focused on the potential of RS to evaluate the margin of bone resection during OSCC mandibular resection (Barroso et al., 2018). They also assessed the water concentrations of healthy and tumor bone tissues (more than 3 mm from the tumor boundary), and a Manne–Whitney U-test revealed significant differences in water concentration between the two groups. Furthermore, they built a PCA-LDA model to distinguish two types of samples based on the intensity of 2800–3050 cm^–1^ C–H stretching. For suspicious spectra proven by water concentration, the PCA-LDA model presented 95% accuracy (sensitivity: 95%, specificity: 87%). By assigning different colors to the tumor bone tissue and normal tissue, they completed a 2D Raman map. Therefore, Barroso et al. believed that RS is a candidate method for intraoperative surgical edge assessment. Compared to the reports from Barroso et al., Cals et al. adopted two-step PCA-(h)LDA to achieve higher accuracy (accuracy: 91%, sensitivity: 100%, specificity: 78%) (Cals et al., 2016). A 2D Raman map can also be constructed using the tumor posterior probability of each sub-site spectrum to present visual information.

RS can also sensitively detect minor changes in a sample, presenting the potential to predict the recurrence rate of oral cancer patients (Sahu et al., 2015; Malik et al., 2017). Carcinoembryonic antigen (CEA) has the potential to predict tumor recurrence, which has been confirmed in other types of cancers (Lumachi et al., 2008). The Raman spectra of sera can detect cancer-related proteins and DNA (Harris et al., 2010). In 2015, Sahu et al. reported that the Raman spectra of sera are related to OSCC recurrence (Sahu et al., 2015). They collected serum samples immediately before and 1 week after surgery. The PCA-LDA classifier could differentiate patients with or without tumor recurrence within 2 years, and the classification effectiveness was approximately 78%. It is worth mentioning that only postoperative serum spectra are related to tumor recurrence. However, serum spectra changes cannot identify the specific location of a tumor, but as a preliminary test to screen high-risk people, they can be effective. Malik et al. applied oral mucosa to predict tumor recurrence. They analyzed the Raman spectra of malignant and contralateral normal mucosa in OSCC patients using PCA-LDA and leave-one-out cross-validation (LOOCV). They focused on misclassification, and found a relationship with recurrence 2 years post-surgery (Malik et al., 2017). They included 57 patients with OSCCs (including tongue, cheek, mandible, molar posterior pad, hard plate, and mouth cancers). Eight of 41 patients with misclassified spectra and 2 of 16 with correct spectra classification had second primary cancer or recurrence 2 years after surgery. It can be concluded that the misclassified group presented 1.5 times greater recurrence risk than the correctly classified group. Tissues have changed at the molecular level before the visible malignant change appears, and Raman spectra can detect this minor transformation. The sensitivity and specificity of the classifier were 80% and 29.7%, respectively. Although the specificity was low, it is acceptable as a preliminary screening tool, and following studies may increase the specificity by increasing the sample size.

## 4 Discussion

Both dental caries and periodontal disease are chronic infectious diseases, in which bacteria and biofilms play a critical role in initiation and progression. Studies have shown that the composition of the subgingival flora and metabolism of virulence factors change significantly before periodontitis advances (Curtis et al., 2020). However, it is not easy to detect changes in the composition and metabolism of flora chairside quickly and precisely. As Raman spectra can sensitively detect changes at the molecular level, they show the potential to detect metabolic changes in bacteria and biofilms chairside. Detailed and comprehensive analysis of the metabolism of bacteria and biofilms by Raman spectra requires interpretation of the meaning and changes of each peak. The amount of information and workload is massive, and it is difficult to reverse the changes at the molecular level from this phenomenon. Focusing on the changes in key bands in the spectra, at this stage, the metabolic changes can be observed more efficiently and intuitively, such as those at 484 cm^–1^ and 2040–2300 cm^–1^ (Tao et al., 2017; Daood et al., 2020a). In addition, there are many reports on the application of Raman spectra to distinguish different oral bacteria, the accuracy of which can be improved by enhancing the spectral signal-to-noise ratio or applying a more distinguishable analysis method.

It is well known that mature laboratory methods such as polymerase chain reaction and western blot can identify bacteria and accurately detect changes in mRNA transcription and protein expression within hours; however, they are more suitable for the laboratory because special reagents and instruments are required. The potential of RS is more inclined to obtaining information in a clinical environment within minutes, with the advantages of being *in situ*, non-invasive, and accurate as RS is not interfered with by water, causes no damage to the sample, and reflects the chemical bond information clearly. To tap its potential in chairside applications, researchers have made many attempts *in vitro*, including identification of bacterial species (Kriem et al., 2020), detecting bacterial metabolic changes through D_2_O-labeled RS (Guo et al., 2019), quorum-sensing molecules (Culhane et al., 2017), and structure of biofilms (Kriem et al., 2021). In addition, as RS can truthfully reflect information on the composition, structure, and concentration of all biological samples, it will provide a basis for obtaining more bacterial information chairside when changes in the flora and the expression of virulence factors can be obtained through RS. However, it should be noted that accurately obtaining target information from a large amount of information still requires study. In terms of hardware foundation, the only portable Raman spectrometer (Thermo Scientific™ Gemini™) is very expensive. Before a unified and standardized inspection process is established, the path to RS chairside inspection is still long. However, RS has undeniably shown strong potential to provide more microbiological information chairside or even *in situ*.

Biological samples are usually composed of proteins, lipids, nucleic acids, and inorganic substances, and various bonds make up the Raman spectra, which often causes confusion over how to extract the desired information. Samples for the diagnosis and prognosis of oral diseases include biofluids (saliva, GCF, and blood/serum), bacteria or cells, soft tissue, and hard tissue (teeth and bone). Gathering bodily fluid samples requires specific conditions and methods to avoid interfering factors. For instance, saliva samples are usually taken at 9:00 to 11:00 a.m. after mouth rinsing one to three times (Gonchukov et al., 2011; Hernández-Cedillo et al., 2019; Yang et al., 2020). Regarding GCF, the paper strips used to take samples must be carefully protected from contamination by blood or saliva, and peripheral blood samples should be taken after 10 h of overnight fasting (Xue et al., 2018). Biofluids are not usually uniformly distributed after drying and often crystallize on solid surfaces, and the spectra obtained at different sites also show large differences (Gonchukov et al., 2011). It is possible to average the spectra obtained from multiple sites for analysis and identification. It is worth mentioning that saliva contains many biomarkers, such as SA (Hernández-Cedillo et al., 2019) and carotenoids (Kim et al., 2010; Gonchukov et al., 2011) for periodontitis diagnosis, and thiocyanate (Falamas et al; Fălămaş et al., 2020) and S100P mRNA (Han et al., 2019) for oral cancer detection.

Planktonic bacteria and cell samples are also unevenly distributed after drying, but bacterial cells are independent as a unit, unlike the uneven distribution of components in bodily fluid samples. Therefore, it is feasible to obtain Raman spectra in combination with a microscope to determine the cell distribution. It should be noted that definite and scientific culture conditions and culture time should be adopted to ensure the stability of the cell state, which is the basis for obtaining stable Raman spectra. After biofilm formation and cell attachment, solid surfaces are formed, and the distribution disappears unevenly. Once samples form a solid plane, Raman maps can be obtained by integrating the intensities of characteristic bonds (such as O–H in high-wavenumber regions) at every site to facilitate the observation of the boundaries of different tissues and provide a basis for judging the edges of tumor tissues (Barroso et al., 2015; Barroso et al., 2016; Barroso et al., 2018). Soft tissues include epithelial, connective, adipose, glandular, and nerve tissues. Some studies have focused on biomarkers and corresponding bonds to differentiate different tissues, such as collagen in gingiva (Garnero et al., 2010; Daood et al., 2018) and keratin in squamous cell carcinoma tissue (Chen et al., 2016). Other studies have analyzed all the information in the spectra, which is complicated. Hard tissues in the oral cavity include teeth (enamel, dentin, and cement) and bone, and changes in phosphate (960 cm^–1^), carbonate (960 cm^–1^), and collagen (1655 or 1667, 1246 or 1270, and 1450 cm^–1^) have attracted attention for detecting caries and defining the edge of decayed tooth tissue (Toledano et al., 2015b). In bone regeneration, OCP and amorphous HAP represent immature bone, and HAP crystals representing mature bone have been used to evaluate bone transformation (Gatin et al., 2019).

There are two ways to assist the diagnosis and prognostic assessment of oral diseases using RS. One is to focus on specific biomarkers that are associated with well-known pathological changes and their corresponding bonds. The advantages of this approach are that the data analysis is simpler and the discrimination efficiency is higher. However, most biomarkers for oral cancer and periodontal disease detection are microRNA, cell-free DNA, extracellular vesicles, and cytokines (interleukin and tumor necrosis factor) (Cristaldi et al., 2019), which do not have specific bonds like SA (Hernández-Cedillo et al., 2019), carotenoids (Kim et al., 2010; Gonchukov et al., 2011), and thiocyanate (Falamas et al; Fălămaş et al., 2020) in saliva. There are several methods to capture target biomarkers and attach labeled molecules to them, but the process is complicated. The other way is to classify the principal components of the full spectra, establish a model, and test its sensitivity and specificity. The most commonly used method for this approach is PCA-LDA+LOOCV. It is worth mentioning that MCR-ALS analysis can decompose complicated spectra into interpretable components, which are accessible to non-specialists in the spectroscopy field (Chen et al., 2016). This is a solution for nucleic acid and protein biomarkers.

RS exhibits unique potential in the biomedical field due to its high sensitivity and accuracy and low water interference and sample damage. However, only a few photons are produced in Raman scattering, the peak intensity is relatively weak, and the signal-to-noise ratio is low. Environmental factors (such as light and vibration) and the parameters selected for Raman spectra acquisition are also interfering factors. The detection sites of unevenly distributed samples (biofluids) and the focus of the microscope also affect the spectra.

The signal-to-noise ratio can be improved during the signal acquisition and data analysis stages. First, combining RS with other technologies can have a positive effect. For example, SERS enhances the local electric field to increase the peak intensity; micro-RS can obtain more accurate information as the signal comes from accurate sites under a microscope, reducing interference signals from other sites; and near-infrared excitation Fourier-transform RS uses Fourier-transform technology to collect signals and accumulate multiple times to improve the signal-to-noise ratio, and irradiates the sample with a 1064 mm near-infrared laser to reduce the fluorescence background. Second, data preprocessing and data analysis can improve the signal-to-noise ratio. The most commonly used data analysis method is PCA-LDA. However, PCA-QDA presents a higher accuracy than PCA-LDA (Jeng et al., 2019). Moreover, the two-step distinguishing process of PCA-(h)LDA has a higher accuracy than the one-step process of PCA-LDA (Cals et al., 2016). MCR-ALS is also an option for non-specialists in the spectroscopy field (Chen et al., 2016). Another user-friendly data analysis method for researchers with limited mathematical knowledge is worth mentioning: Inverted Discrete Wavelet Transform decomposes the Raman spectra into low-frequency (approximation) components and fluctuation (detail) components. The next layer of “approximation” and “detail” components is continuously decomposed from the previous “approximation” components. The last “approximation” and several previous “detail” components are finally integrated to filter out non-correlated signals and background signals, improving the readability of the Raman spectra (Camerlingo et al., 2008).

Third, focusing on biomarkers, such as keratin in tumor tissue (Chen et al., 2016), SA (Hernández-Cedillo et al., 2019), carotenoids (Kim et al., 2010; Gonchukov et al., 2011), thiocyanate (Falamas et al; Fălămaş et al.), and S100P mRNA (Han et al., 2019), also increases the differentiation accuracy. Subcellular DNA (Carvalho et al., 2017) and DNA information from dehydrated cells (Panikkanvalappil et al., 2013) also provide more valid information.

Collectively, RS has shown its ability to assist in the diagnosis and prognostic prediction of oral diseases. Its high sensitivity and accuracy and low water interference and sample damage are useful for obtaining information from not only isolated samples but also *in situ* sites, such as in detection of early caries and malignant lesions of, e.g., oral mucosa, *in vivo.* However, the collection of spectra with higher signal-to-noise ratios and selection of better data analysis methods to achieve higher accuracy are still the focus of further studies and the direction of future efforts.

## Author Contributions

YZ and LR drafted the manuscript. QW and ZW provided valuable insights for the manuscript. CL and YD reviewed and edited the manuscript. All authors have approved the final version of the manuscript.

## Funding

This study was supported by the National Natural Science Foundation of China (grant number 82071121 to YD), Open Project of State Key Lab of Optical Technologies on Micro-Engineering and Nano-Fabrication (2021LF1007), and Sichuan University crosswise task (21H0674).

## Conflict of Interest

The authors declare that the research was conducted in the absence of any commercial or financial relationships that could be construed as a potential conflict of interest.

## Publisher’s Note

All claims expressed in this article are solely those of the authors and do not necessarily represent those of their affiliated organizations, or those of the publisher, the editors and the reviewers. Any product that may be evaluated in this article, or claim that may be made by its manufacturer, is not guaranteed or endorsed by the publisher.

## Abbreviations

SERS, surface-enhanced RS; AuNPs, Au nanoparticles; AgNPs, Ag nanoparticles; FWHM, full width at half maximum; BIMIN, biomimetic mineralization kit; QAS, quaternary ammonium salts; D_2_O, heavy water; WSL, white spot lesion; BAG, bioactive glass; PAA-BAG, BAG containing polyacrylic acid; PCA, principal component analysis; GCF, gingival crevicular fluid; SA, sialic acid; @IL-1β, interleukin-1β; TNF-α, tumor necrosis factor-α; DTNB, 5,5’-Dithiobis-(2-nitrobenzoic acid); 4-MBA, 4-mercaptobenzoic acid; OCP, octacalcium phosphate; HAP, hydroxyapatite; OSCC, oral squamous cell carcinoma; TNM classification, primary tumor, regional lymph nodes, and metastasis classification; WHO, World Health Organization; OSMF, oral submucous fibrosis; OLK, leukoplakia; OPL, oral precancerous lesion; HT, healthy tobacco users; HV, healthy volunteers; PCA-LDA, principal component analysis with linear discriminant analysis; PCA-LR, principal component analysis with logistic regression; FTIR, Fourier-transform infrared spectroscopy; SVM, support vector machine; LDA, linear discriminant analysis; QDA, quantitative discrimination analysis; PCA-QDA, principal component analysis with quantitative discrimination analysis; PCA-(h)LDA, principal component analysis with (hierarchical) linear discriminant analysis; MCR-ALS, multivariate curve resolution with alternating least squares; S100P, S100 calcium-binding protein P; LOOCV, leave-one-out cross-validation.
